# Expanding TAVI to Low and Intermediate Risk Patients

**DOI:** 10.3389/fcvm.2018.00092

**Published:** 2018-07-12

**Authors:** Lisa Voigtländer, Moritz Seiffert

**Affiliations:** ^1^Department of General and Interventional Cardiology, University Heart Center Hamburg, Hamburg, Germany; ^2^Partner site Hamburg/Kiel/Lübeck, DZHK, German Centre for Cardiovascular Research, Hamburg, Germany

**Keywords:** TAVI, TAVR, intermediate risk, low risk, aortic valve stenosis

## Abstract

TAVI has become the standard treatment in patients at increased surgical risk and is increasingly being performed in patients at intermediate to low surgical risk. While non-inferiority has been demonstrated in intermediate risk patients, several challenges—particularly with regard to valve durability—need to be addressed before expansion to lower risk and younger patients can be recommended on a broad basis. Current trends, trials results, and remaining challenges are summarized and discussed in the light of updated treatment guidelines.

## Introduction

**Figure 1:**
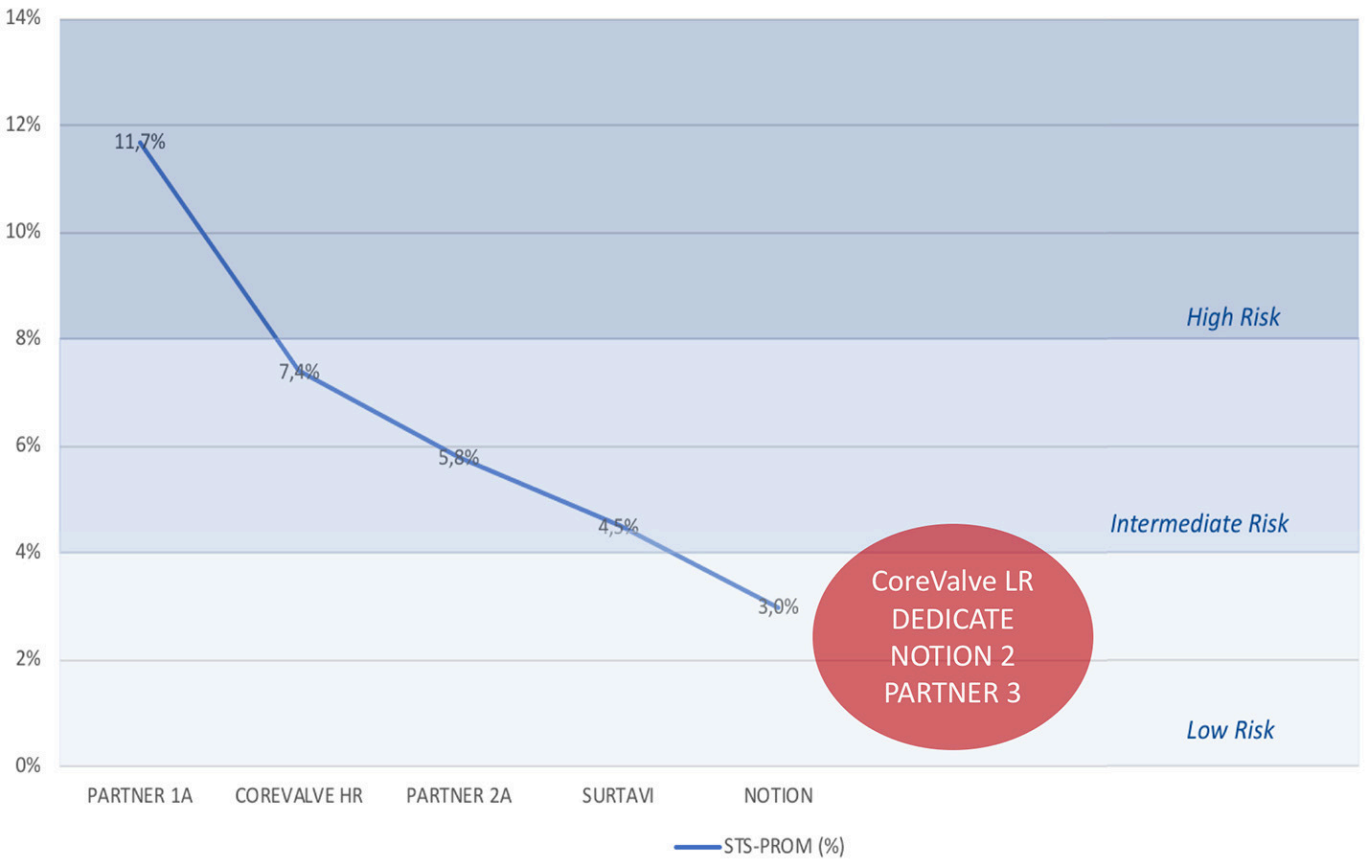
Evolution of operative risk in major trials. Decline in operative risk, as assessed by the STS-PROM score, in major randomized trials comparing TAVI and SAVR (6–10) and anticipated low to intermediate risk of currently active trials (CoreValve LR, DEDICATE, NOTION 2, PARTNER 3). SAVR, surgical aortic valve replacement; STS-PROM, Society of Thoracic Surgeons' Predicted Risk of Operative Mortality; TAVI, transcatheter aortic valve implantation.

Severe aortic valve stenosis (AS) represents the most common valvular heart disease in developed countries. Since its prevalence is associated with increasing age, a growing disease burden is expected in the future considering an aging patient population (1). Surgical aortic valve replacement (SAVR)—the traditional standard of care for patients with severe symptomatic AS—is increasingly complemented by transcatheter aortic valve implantation (TAVI). After the first TAVI procedure in 2002 (4), the number of procedures has increased exponentially in the past years and has recently outperformed the number of isolated SAVR per year in Germany (5). Several prospective randomized trials demonstrated non-inferiority for TAVI compared to SAVR in patients at high surgical risk (6, 7). More recently, three additional trials reported non-inferiority of TAVI in intermediate-risk patients (Figure 1, Table 1) (8–10). Current debates focus on the expansion of TAVI as the standard of care for the treatment of patients with AS and low to intermediate operative risk.

**Table 1:** Results of major prospective randomized trials on TAVI vs. SAVR in high and intermediate to low risk patients.

	**PARTNER 1A** (6)			**CoreValve HR** (7)			**PARTNER 2A** (10)			**NOTION** (9)			**SURTAVI** (8)		
**Time of recruitment**	**May 2007–August 2009**			**February 2011–December 2012**			**December 2011 –November 2013**			**December 2009–April 2013**			**June 2012 –June 2016**		
**THV**	**SAPIEN**			**CoreValve**			**SAPIEN XT**			**CoreValve**			**CoreValve**		
**Primary endpoint**	**All-cause death at 1 year**			**All-cause death at 1 year**			**All-cause death or diasbling stroke at 2 years**			**All-cause death, disabling stroke or myocardial infarction at 1 year**			**All-cause death or disabling stroke at 2 years**		
	**TAVI**	**SAVR**	**p**	**TAVI**	**SAVR**	**p**	**TAVI**	**SAVR**	**p**	**TAVI**	**SAVR**	**p**	**TAVI**	**SAVR**	**95% CI**
Number of randomized pts.	348	351	n/a	394	401	n/a	1,011	1,021	n/a	145	135	n/a	864	796	n/a
Age	83.6 ± 6.8	84.5 ± 6.4	0.07	83.2 ± 7.1	83.5 ± 6.3	n/a	81.5 ± 6.7	81.7 ± 6.7	n/a	79.2 ± 4.9	79.0 ± 4.7	n/a	79.9 ± 6.2	79.7 ± 6.1	n/a
Male gender	57.8%	56.7%	0.82	53.6%	52.9%	n/a	54.2%	54.8%	n/a	53.8%	52.6%	n/a	57.6%	55.0%	n/a
STS-PROM (%)	11.8 ± 3.3	11.7 ± 3.5	0.61	7.3 ± 3.0	7.5 ± 3.2	n/a	5.8 ± 2.1	5.8 ± 1.9	n/a	2.9 ± 1.6	3.1 ± 1.7	n/a	4.4 ± 1.5	4.5 ± 1.6	n/a
Log. EuroSCORE (%)	29.3 ± 16.5	29.2 ± 15.6	0.93	17.6 ± 13.0	18.4 ± 12.8	n/a	n/a	n/a	n/a	8.4 ± 4	8.9 ± 5.5	n/a	11.9 ± 7.6	11.6.8.0	n/a
Transfemoral access	70.1%	–	–	n/a	–	–	76.3%	–	–	96.5%	–	–	93.6%	–	–
Primary endpoint	24.2%	26.8%	0.44	14.2%	19.1%	0.04	19.3%	21.1%	0.25	13.1%	16.3%	0.43	12.6%	14.0%	−5.2 to 2.3
All-cause mortality (30 days)	3.4%	6.5%	0.07	3.3%	4.5%	n/a	3.9%	4.1%	0.78	2.1%	3.7%	0.43	2.2%	1.7%	−0.9 to 1.8
All-cause mortality (1 year)	24.2%	26.8%	0.44	14.2%	19.1%	0.004	12.3%	12.9%	0.69	4.9%	7.5%	0.38	6.7%	6.8%	−2.7 to 2.4
All-cause mortality (2 years)	33.9%	35.0%	0.78	22.2%	28.6%	0.04	16.7%	18.0%	0.45	8.0%	9.8%	0.54	11.4%	11.6%	−3.8 to 3.3
Stroke (30 days)	3.8%	2.1%	0.2	4.9%	6.2%	0.46	5.5%	6.1%	0.57	1.4%	3.0%	0.37	3.4%	5.6%	−4.2 to−0.2
Myocardial infarction (30 days)	0	0.6%	0.16	0.8%	0.8%	0.92	1.2%	1.9%	0.22	2.8%	6.0%	0.2	0.9%	1.0%	−1.0 to 0.9
Major vascular complications (30 days)	11.0%	3.2%	<0.001	5.9%	1.7%	0.003	7.9%	5.0%	0.008	5.6%	1.5%	0.1	6.0%	1.0%	3.2 to 6.7
Major or life-threatening bleeding (30 days)	9.3%	3.2%	<0.001	13.6%	35.0%	<0.001	10.4%	43.4%	<0.001	11.3%	20.9%	0.03	12.2%	9.3%	−0.1 to 5.9
New permanent pacemaker (30 days)	3.8%	3.6%	0.89	19.8%	7.1%	<0.001	8.5%	6.9%	0.17	34.1%	1.6%	<0.001	25.9%	6.6%	15.9–22.7
New-onset atrial fibrillation (30 days)	8.6%	16.0%	0.006	11.7%	30.5%	<0.001	9.1%	26.4%	<0.001	16.9%	57.8%	0.001	12.9%	43.3%	−34.7 to −26.4
Acute kidney injury (30 days)	1.2%	1.2%	0.95	6.0%	15.1%	<0.001	1.3%	3.1%	0.006	0.7%	6.7%	0.01	1.7%	4.4%	−4.4 to −1.0
Moderate or severe PVL (1 year)	6.8%	1.9%	<0.001	6.1%	0.5%	<0.001	3.4%	0.4%	<0.001	15.7%	0.9%	0.001	5.3%	0.6%	n/a
Rehospitalization (2 years)	24.7%	21.7%	0.41	n/a	n/a	–	19.6%	17.3%	0.22	n/a	n/a	–	13.2%	9.7%	0.1–7.0
Endocarditis (2 years)	1.5%	1.0%	0.61	0.9%	1.7%	0.35	1.2%	0.7%	0.22	n/a	n/a	–	n/a	n/a	–
AV reintervention (2 years)	n/a	n/a	–	2.5%	0.4%	0.02	1.4%	0.6%	0.09	n/a	n/a	–	2.7%	0.7%	0.6–3.4

*AV, aortic valve; CI, confidence interval; LogES, logistic EuroSCORE; PVL, paravalvular leakage; SAVR, surgical aortic valve replacement; STS-PROM, Society of Thoracic Surgeons predicted risk of operative mortality; TAVI, transcatheter aortic valve intervention; THV, transcatheter heart valve. Values are given as mean ± standard deviation or frequencies and percentages*.

## Assessment of operative risk

What are criteria and cutoffs for low to intermediate operative risk? Objective risk estimation remains the Achilles' heel for the evaluation of individual treatment options and overall comparison of clinical trial results. A multitude of relevant clinical and anatomical factors effectively influence operative complexity, complicating precise risk calculation in these patients. All of the widely-used risk stratification tools (STS-PROM, logistic EuroSCORE, EuroSCORE II) entail significant limitations in predicting operative mortality (11, 12). In the absence of a perfectly reliable risk model, the STS-PROM has mostly been applied for individual risk assessment and for comparison of trials results. In the past, operative risk was classified as high (STS-PROM >8%), intermediate (STS-PROM of 4–8%), and low (STS-PROM <4%). However, important additional factors, e.g. active malignancy, frailty, porcelain aorta, chest wall radiation, liver cirrhosis, or neurological impairment, were not comprehensively integrated in these risk models. In addition, treatment decisions may differ in elderly patients without comorbidities (low operative risk despite advanced age) and young patients with significant comorbidities (increased operative risk despite young age). The 2017 ESC/EACTS guidelines for the management of valvular heart disease incorporate these difficulties and opt for a more differentiated approach to operative risk and choice of treatment modality (13).

## Treatment selection according to current guidelines

To help navigate the choice of treatment modality in patients with low to intermediate surgical risk, European (13) and American (14) guidelines were recently updated. In general, the indication for TAVI was expanded to intermediate risk patients in both versions on the basis of three major trials (8–10). American guidelines in its current version consider TAVI a reasonable alternative to SAVR in patients at intermediate operative risk (STS-PROM ≥4%), depending on patient-specific procedural risks, values, and preferences (14). European guidelines emphasize, that the treatment selection (TAVI or SAVR) in patients at increased surgical risk (STS-PROM ≥4%, logistic EuroSCORE ≥10% or risk factors not considered in these algorithms) should be made by the Heart Team on an individualized basis (13). According to the guideline's authors, factors in favor of a catheter-based approach include patient age ≥75 years, prior cardiac surgery, frailty, restricted mobility or anticipated prolonged rehabilitation, favorable transfemoral access, prior chest radiation, porcelain aorta, severe chest deformation, or expected prosthesis-patient mismatch. Other aspects, e.g. patient age <75 years, suspicion of endocarditis, unfavorable anatomy for TAVI (access, low coronary take-off, unfavorable aortic root, valvular, or annular anatomy), and concomitant cardiac conditions that require additional surgical treatment favor SAVR. Overall, SAVR remains the standard therapy for patients <75 years of age with low surgical risk at current as long-term durability data for THV remain insufficient. In the absence of a perfect risk assessment, both guidelines emphasize the integral role of the interdisciplinary heart team in patient evaluation, assessment of technical suitability, and identification of the appropriate treatment modality (13, 14).

## Evidence from intermediate-risk trials or registries

Essential evidence for the expansion of TAVI for the treatment of intermediate risk patients stems from three prospective randomized trials and reports from major contemporary registries.

### Registries

Several large-scale nationwide registries evaluated outcomes and trends in the treatment of aortic valve stenosis. Long before first results from prospective randomized intermediate-risk trials were available, large registries had already reported a paradigm shift of TAVI towards lower risk patients: According to the compulsory German quality assurance registry on aortic valve replacement (AQUA), the number of annual TAVI procedures in Germany increased 20-fold from 2008 to 2014 while the number of SAVR procedures slowly declined (15). Interestingly, operative risk, as assessed by the logistic EuroSCORE, decreased significantly over the years with a larger percentage of patients at low to intermediate risk in the later years (logES<10%: 18.9% [2012] vs. 25.9% [2014]). This was followed by a drop in hospital mortality after TAVI during the observation period (2008: 10.4%, 2014: 4.2%) (15).

Similar trends were observed in the German Aortic Valve Registry (GARY), which included a total of 15,964 patients undergoing TAVI between 2011 and 2013 (16). Over the years, a significant regression in risk profiles (logES 20.2% [2011] to 16.9% [2013]; STS-PROM: 5.2% [2011] to 4.9 [2013], both *p* < 0.001), periprocedural complications and in-hospital mortality (5.9% [2011] to 4.9% [2013], *p* = 0.078) were observed (16).

The Society of Thoracic Surgeons (STS)/American College of Cardiology Transcatheter Valve Therapy (TVT) Registry collected data from 54,782 TAVI procedures performed in the United States from 2012 to 2015. The volume of annual TAVI procedures increased from 4,627 to 24,808 in this time window (17). While the median STS-PROM decreased from 7.1 to 6.3% (2012 vs. 2015, *p* < 0.001), a subsequent decline of 30-day mortality (7.5% [2012] vs. 4.6% [2015], *p* < 0.0001), stroke (2.3% [2012] vs. 1.9% [2015], *p* = 0.0264), or moderate/severe PVL (2012:10.8% [2012] vs. 6.2% [2015], *p* < 0.0001) was observed (17).

A shift in patients' disease severity and advancements in procedural and technical aspects over the past years have most likely contributed to these consistent improvements of outcomes after TAVI. However, a comparison of treatment modalities from these registries' results is impeded by very different risk profiles in the treatment groups, calling for appropriate randomized trials.

### Randomized trials

In addition to several real-world registries, few comprehensive—but highly selective—industry-sponsored trials evaluated outcomes after TAVI in different risk categories (see Table 1 for selected results, Figure for risk profile). Results of intermediate risk trials are discussed in the following.

The first randomized trial to evaluate TAVI in low to intermediate risk patients was the Nordic STACCATO trial. It started patient recruitment as early as 2008 and aimed to compare transapical TAVI to SAVR in operable patients ≥75 years of age (18). Due to an excess of serious adverse events in the transapical TAVI arm, the study was prematurely terminated after inclusion of 70 patients. The trial was heavily criticized for its design, including only a transapical TAVI arm.

One year later, the NOTION (Nordic Aortic Valve Intervention) trial (9) started recruitment. NOTION randomized 280 patients ≥70 years of age with severe aortic stenosis to TAVI with the Medtronic CoreValve THV or SAVR at three Nordic centers (TAVI:145 patients; SAVR: 135 patients). Mean STS-PROM was 2.9 ± 1.6% in TAVI and 3.1 ± 1.7% in SAVR patients. The access route was transfemoral in 96.5% of TAVI cases. The composite primary endpoint (all-cause mortality, stroke or myocardial infarction) and all-cause mortality were similar in both groups (13.1% [TAVI] vs. 16.3% [SAVR] and 4.9% [TAVI] vs. 7.5% [SAVR], *p* = 0.38). Periprocedural complications differed according to treatment arm with an access of major/life-threatening bleeding (11.3% [TAVI]) vs. 20.9 [SAVR]), acute kidney injury stage 3 (0.7% [TAVI] vs. 6.7% [SAVR]), and new-onset or worsening atrial fibrillation (16.9 [TAVI] vs. 57.8% [SAVR], *p* < 0.001) in the SAVR arm. Rates of permanent pacemaker implantation (34.1% [TAVI] vs. 1.6% [SAVR], *p* < 0.001) and PVL (moderate/severe at 1 year: 15.7% [TAVI] vs. 0.9% [SAVR]) were observed more frequently in patients treated with TAVI. At the same time, transvalvular gradients and effective orifice areas were in favor of TAVI treatment. Recent 5-year data confirmed non-inferiority of TAVI compared to SAVR regarding the composite endpoint (TAVI: 39.2%; SAVR 35.8%; *p* = 0.78) (2) and the 5-year all-cause mortality of 27.7% was the lowest 5-year mortality rate ever reported in a TAVI population. NOTION was the first prospective randomized trial to generate data on TAVI in intermediate to low risk patients. However, the small sample size and the large rate of screening failures challenge the “all-comers” character of the trial.

At a larger scale, the PARTNER 2A trial randomized 2,032 patients with intermediate surgical risk (STS-PROM score 4–8% and heart team consensus) to either TAVI with the balloon-expandable SAPIEN XT or SAVR (10). The mean STS-PROM was 5.8% and almost twice as high as in the NOTION trial. The composite endpoint at 2 years (all-cause death or disabling stroke) was non-inferior in patients treated with TAVI compared to SAVR (TAVI: 19.3%, SAVR: 21.1%, *p* = 0.25). A subsequent subgroup analysis even demonstrated superiority for the transfemoral cohort compared to SAVR (16.3 vs. 20%, *p* = 0.04). At 2 years of follow-up, a higher incidence of life-threatening/disabling bleeding (47.0 vs. 17.3%, *p* < 0.001), acute kidney injury stage 3 (6.2 vs. 3.8%, *p* = 0.02), and new onset atrial fibrillation (27.3 vs. 11.3%, *p* < 0.001) were reported after SAVR while patients after TAVI had a higher risk for major vascular complications (8.6 vs. 5.5%, *p* = 0.006). Interestingly, rates of permanent pacemaker implantations were not significantly different in both groups in this trial. An overall faster recovery and shorter hospitalization (in-hospital: median 6 vs. 9 days, ICU: median 2 vs. 4 days, *p* < 0.001 for both) were observed after TAVI. While lower transprosthetic gradients were reported in the TAVI arm, the rate of moderate/severe PVL was significantly higher compared to SAVR (8.0 vs. 0.6%, *p* < 0.001) and a trend towards more aortic valve re-interventions was observed after TAVI at 2 years (1.4 vs. 0.6%, *p* = 0.09). This observation has to be followed closely as the TAVI indication is expanded to younger patients. Of note, 14.5% of patients in the SAVR arm underwent concomitant coronary artery bypass graft surgery for significant coronary artery disease.

After a recruitment period of almost 4 years, the SURTAVI trial (8) recently reported results of 1,764 patients at intermediate surgical risk (predicted 30-day operative mortality 3–15%). The mean STS-Score was 4.5 ± 1.6% and thus in between the PARTNER 2A and NOTION trials. Patients were randomized 1:1 to TAVI with the self-expanding CoreValve or CoreValve Evolut R prostheses and SAVR. The primary endpoint, a composite of all-cause death and disabling stroke at 2 years, was similar in both treatment arms (12.6% [TAVI] vs. 14% [SAVR], 95%CI −5.2 to 2.3%). Again, higher rates of acute kidney injury (4.4 vs. 1.7%), new onset atrial fibrillation (43.4 vs. 12.9%), and transfusion requirements (41.1 vs. 12.5%) were observed after SAVR. While hemodynamic measures were in favor of TAVI (transprosthetic gradients, effective orifice area), the incidence of PVL (moderate/severe at 1 year: 5.3 vs. 0.6%) and the need for pacemaker implantation (25.9 vs. 6.6%) were lower after SAVR. Quality of life at 2 years was similar in both groups. Aortic valve reintervention was reported more often after TAVI (2.7 vs. 0.7% at 2 years), although no structural valve deterioration was found in either group.

### Currently active intermediate to low risk trials

Building on the results of intermediate-risk trials and registries named above, several prospective randomized trials are currently active, either recruiting patients or in follow-up, to evaluate outcomes after TAVI in patients at low to intermediate operative risk. The results of these trials will determine future guideline recommendations on the treatment of aortic stenosis in low to intermediate risk patients (see Table 2 for major characteristics of these trials).

**Table 2:** Overview of currently active randomized trials on TAVI vs. SAVR in low to intermediate risk patients with severe aortic stenosis.

	**DEDICATE**	**NOTION 2**	**PARTNER 3**	**CoreValve low risk**
Reference/NCT number	Clinicaltrials.gov/NCT03112980	Clinicaltrials.gov/NCT02825134	Clinicaltrials.gov/NCT02675114	Clinicaltrials.gov/NCT02701283
Study start date	2017	2016	2016	2016
Study status	Recruiting	Recruiting	Recruiting	Recruiting
Estimated study completion date	2024	2024	2027	2026
Patients' risk profile	STS-PROM 2-6%	Patient age ≤75 years and STS-PROM <4%	STS-PROM <4%	Operative risk <3%
Study arms	TAVI^*^ vs. SAVR^*^ (1:1 randomization)	TAVI^*^ vs. SAVR^*^ (1:1 randomization)	TAVI (SAPIEN 3) vs. SAVR^*^ (1:1 randomization)	TAVI (CoreValve Evolut R) vs. SAVR^*^ (1:1 randomization)
Estimated enrollment	1,600	992	1,328	1,200
Primary Outcome	Efficacy endpoint: Overall survival at 5 years	All-cause mortality, myocardial infarction or stroke at 1 year	All-cause mortality, stroke, or re-hospitalization at 1 year	All-cause mortality or disabling stroke at 2 years
	Safety endpoint: Overall survival at 1 year and 196 deaths (event-driven)			
Follow up time	5 years	1 year	10 years	10 years
Listed location countries	Germany	Denmark, Finland, Iceland, Norway, Sweden	Australia, Canada, Japan, New Zealand, United States	Australia, Canada, France, Netherlands, New Zealand, Switzerland, United States
Study sponsor and collaborators	University Medical Center Hamburg-Eppendorf	Rigshospitalet, Denmark Symetis SA, Boston Scientific Corporation, St. Jude Medical	Edwards Lifesciences	Medtronic Cardiovascular
	German Center for Cardiovascular Research (DZHK)			

SAVR, surgical aortic valve replacement; STS-PROM, Society of Thoracic Surgeons predicted risk of operative mortality; TAVI, transcatheter aortic valve intervention;

* *Any commercially available or CE marked device. Information up-to-date as available on clinicaltrials.gov on June 10th, 2018*.

The PARTNER 3 trial (clinicaltrials.gov NCT02675114) randomly assigns 1,328 patients with low surgical risk (STS-PROM <4%) to TAVI with the Sapien 3 device or SAVR. Patients will be followed for 10 years and the primary endpoint is a composite of all-cause mortality, stroke and rehospitalization at 1 year. Results of the primary endpoint are expected to be presented in 2019.

The Medtronic TAVR low risk trial (clinicaltrials.gov NCT02701283) includes 1,200 patients with an STS-PROM <3%. Patients are randomized to TAVI with the CoreValve or CoreValve Evolut R self-expandable THV or SAVR. Patients will be followed for 10 years and the primary endpoint is a composite of all-cause mortality or disabling stroke at 2 years.

While both studies are industry-sponsored and limited to one THV, two additional investigator-initiated trials have been initiated:

The Nordic NOTION-2 trial (clinicaltrials.gov NCT02825134) aims to randomize 992 low risk patients (STS <4%, ≤75 years) to TAVI with any CE-marked device or SAVR. Due to the exclusion of elderly patients, this trial will particularly gain important insights into outcomes of TAVI in younger patients at low risk. Interestingly, combined procedures (SAVR and concomitant CABG or TAVI and PCI) are also included in the trial. The primary endpoint is a composite of all-cause mortality, stroke or myocardial infarction at 1 year. The trial is investigator-initiated but industry-funded.

The DEDICATE trial (DEDICATE-DZHK6, clinicaltrials.gov NCT03112980) is multicenter investigator-initiated and industry-independent study. It is funded by the DZHK (German Center for Cardiovascular Research), the Deutsche Herzstiftung e.V., and supported by German health insurance providers. Overall 1,600 patients at low to intermediate surgical risk (STS-PROM 2–6%) will be included. As opposed to previous trial designs, DEDICATE aims to investigate a true all-comers patient population and evaluate real-world outcomes. After 1:1 randomization to either TAVI or SAVR, the remaining treatment decisions (e.g. access route, THV type, periprocedural treatment, etc.) are left to the interdisciplinary heart team. All CE-marked devices can be utilized to avoid any potential device-based bias. To account for the increasing importance of long-term data in low risk patients, the primary endpoint was chosen as overall survival after 5 years. Low to intermediate risk patients undergoing aortic valve treatment at the study sites who are not included in the randomized trial will be captured in a nested registry to evaluate an all-comers population.

All of these active trials will add significantly to the current evidence for TAVI in intermediate to low risk patients and allow first insights into long-term results on a broad basis.

## Remaining challenges

Within the last decade, TAVI has become the standard of care for high-risk patients with severe and symptomatic AS. It has increasingly been performed in intermediate and also low-risk patients more recently. Particularly for younger and low-risk patients, additional challenges need to be addressed:

### Valve durability and function

The unresolved issue of long-term valve durability is probably the key challenge in expanding TAVI to lower risk and younger age patients. Longitudinal echocardiographic evaluation of the PARTNER trials (PARTNER 1A, 1B, and continued access) demonstrated stable hemodynamic results after TAVI over 5 years of follow-up (19). Similar results were reported in other series and for self-expanding transcatheter heart valves (20). Recently results from the Nordic NOTION trial confirmed not only robust hemodynamic data over 5 years of follow-up but also favorable hemodynamics after TAVI compared to SAVR (2). Particularly in patients with smaller aortic annuli, TAVI may yield a lower incidence of patient-prosthesis mismatch, compared to SAVR. However, increased rates of PVL were consistently observed after TAVI compared to SAVR. Due to an adverse effect of significant paravalvular leakage on survival (10), reduction of residual regurgitation will be essential to improve long-term outcomes. Although progress has been made to reduce residual AR after TAVI in recent studies with next-generation devices (21), further improvements will be required to match data from SAVR cohorts.

Additionally, subclinical leaflet thrombosis, its effects on hemodynamic and clinical results need to be evaluated due to a significantly higher incidence after TAVI compared to SAVR (22). Overall, the incidence of structural valve degeneration and aortic valve re-intervention were low but will naturally become an issue as follow-up length and patient numbers increase. Recently published definitions of prosthesis degeneration may aid comprehensive analysis of this important topic (23, 24). To eliminate durability concerns after TAVI, very solid durability data available for surgical bioprostheses over the course of more than a decade will need to be matched (25).

Nevertheless, degeneration of THV will occur at some point in patient life, leading to either surgical valve replacement or valve-in-valve procedures. Valve-in-valve procedures have demonstrated encouraging results in patients with degenerated surgical aortic bioprostheses (26). Whether these results can be systematically achieved for valve-in-valve procedures in degenerated THV needs to be demonstrated. Different design features of THV may yield variable results after valve-in-valve implantation, for example with regard to coronary access in degenerated supra-annular THV.

While moving towards younger patients, the prevalence of biscuspid aortic valve disease will inevitably increase. Data from retrospective registries demonstrated lower procedural success and higher residual PVL after TAVI in patients with bicuspid compared to tricuspid aortic valve disease (27–30). Implantation of new-generation devices yielded improved outcomes, giving rise to hope that TAVI may become a valid treatment option in bicuspid aortic valve disease in the future (30). Due to the paucity of data, guidelines favor SAVR in these patients at current (13).

### Morbidity and mortality

After early reports of increased stroke rates after TAVI (6), more recent trials have consistently demonstrated similar outcomes for mortality and stroke after TAVI or SAVR. However, distinct complication patterns have repeatedly been reported for both treatment options (see Table 1). These need to be weighed against the individual patient's risk profile when choosing the optimal treatment modality. These include a higher incidences of acute kidney injury, bleeding events, and atrial fibrillation after SAVR. TAVI was associated with faster recovery and shorter index hospitalization but a higher rate of re-interventions or heart failure were documented during follow-up. Long-term results will be essential to gain further insights into these important first observations. While major vascular complications were common after transfemoral TAVI with first-generation devices (31), a significant decrease was observed in recently reported intermediate-risk trials (8–10). A shift in patients' risk and device refinements with smaller delivery systems and improved vascular closure devices may be responsible for this decline. Permanent pacemaker implantation remains a concern after TAVI, particularly with self-expanding THV. Although data remain ambiguous regarding the association of pacemaker implantation and outcome after TAVI at current (32, 33), this issue requires in-depth evaluation, particularly in the treatment of younger patients.

Although a major advantage of TAVI relates to the less invasive procedure compared to SAVR, the risk for rare but life-threatening complications after TAVI (e.g., annular rupture, valve migration, or coronary obstruction) requiring bail-out emergency cardiac surgery must be taken into account. Recently published data from the European Registry on Emergent Cardiac Surgery during TAVI (EuRECS-TAVI) reported an incidence of emergent cardiac surgery of 0.7% in recent years. Most common causes were left ventricular guidewire perforations (28.3%) and annular ruptures (21.2%). Most of these complications occurred during the procedure and mortality remained high despite emergent cardiac surgery (34). While these serious procedure-related complications were more frequent in the early TAVI era and have become very rare events at this stage (35), expansion of TAVI towards younger and low-risk patients requires an even more critical appraisal and all measures need to be taken to prevent these complications.

### Cost-effectiveness

With the rapid growth of TAVI volume, its implications on healthcare systems and its cost-effectiveness will become even more important, particularly while expanding TAVI indications to lower risk patients (36). An early analysis from the Netherlands demonstrated higher 1-years costs of TAVI vs. SAVR in intermediate-risk patients (37). This cost difference was mainly driven by the difference in device prices. A recent cost-effectiveness analysis from the Partner 2A and Sapien 3 trials reported lower costs at 2 years after TAVI (3). Higher procedural costs were compensated for by shorter hospitalization and substantially lower costs during follow-up. Regional and national differences in reimbursement and device costs impede generalization of these results. However, health economic analyses will gain importance as the field expands.

## Conclusion

TAVI has become the standard treatment in patients at increased surgical risk and is increasingly being performed in patients at intermediate to low risk at current. Non-inferiority has been demonstrated in different intermediate risk cohorts. However, before broad expansion to lower risk and younger patients can be recommended, several challenges—particularly with regard to valve durability—need to be addressed. Several randomized trials are under way to investigate these issues and will determine future guideline recommendations. For now, distinct risks should be weighed into the decision of TAVI vs. SAVR, incorporating each patient's individual risk profile and personal preferences. Shared-decision making will increasingly become a crucial element in this process. Preferences of the informed patient should be discussed, balanced, and weighed into the joint treatment decision of the interdisciplinary heart team to select the appropriate treatment for every individual patient while expanding TAVI to intermediate and low risk operative patients.

## Author contributions

LV: literature research, drafted the manuscript; MS: literature research, critical revision of the manuscript.

### Conflict of interest statement

MS received travel compensation from Edwards Lifesciences and lecture fees from Medtronic, serves as coordinating investigator for the DEDICATE-DZHK6 trial. The remaining author declares that the research was conducted in the absence of any commercial or financial relationships that could be construed as a potential conflict of interest.

## Abbreviations

AS: Aortic stenosis
LogES: Logistic EuroSCORE
PVL: Paravalvular leakage
SAVR: Surgical aortic valve replacement
STS-PROM: Society of Thoracic Surgeons' Predicted Risk of Operative Mortality
TAVI: Transcatheter aortic valve implantation
THV: Transcatheter heart valve.
